# Welcoming talent? A comparative study of immigrant entrepreneurs’ entry policies in France, Germany and the Netherlands

**DOI:** 10.1186/s40878-018-0092-4

**Published:** 2018-09-18

**Authors:** Tesseltje de Lange

**Affiliations:** University of Amsterdam, Faculty of Law, Amsterdam Research Institute in Legal Studies (ARILS), Roeterseilandcampus, Building A room 9.05, Nieuwe Achtergracht 166, Amsterdam, 1018 WV The Netherlands

**Keywords:** Immigrant entrepreneurs, Highly skilled migration, National economic interest, Migration law, Comparative law

## Abstract

This article explores the admission policies for self-employed non-EU immigrants wanting to start or move their business to the European Union (EU). Selecting immigrant entrepreneurs is a specific and understudied policy strand in the battle for talent. No common EU policy is available (yet) and although national policies do show some similarity, they differ in respect of how and who decides if an entrepreneur serves a national economic interest. By presenting a first-time model for defining the level of welcoming, this study adds an instrument to the toolbox of both scholars and policy makers for evaluating immigration policies. Whether a policy is welcoming depends on material criteria, such as entry conditions giving the entrepreneur a fair chance and on the formal criteria of the applicable procedures and the actors involved in the decision-making process. The body of the article constitutes of a legal comparison between French, German and Dutch entry policies for non-EU entrepreneurs. The article concludes that a future EU policy on welcoming immigrant entrepreneurs must set standards for a large variety of entrepreneurs, allow for the economic interest to be broadly defined and have, at the least, transparent and practical procedures.

## Introduction

Although immigrant entrepreneurs have received plenty of academic attention over the past decades (Beckers & Blumberg, 2013; Kloosterman, 2010; Kloosterman & Rath, 2003; Mahuteau, Piracha, Tani, & Vaira-Lucero, 2011; Shahin, Nijkamp, & Suzuki, 2014; Solano, 2016), the admission policies for non-EU, also called Third Country National (TCN) self-employed workers or entrepreneurs into EU member states is a rather understudied topic. In recent years academic attention has been paid to these admission policies, but with a strong focus on investor visas for the ‘super rich’ (Dzankic, 2015; Surak, 2016; Torkian, 2015). With the OECD (2014, 2016) call for more labor migration from outside the EU and the increased flexibilization on many European labor markets, it’s likely not just migrant employees, but also self-employed migrants that are to fill future labor market needs. This possible increasing need for welcoming TCN entrepreneurs calls for research attention into this policy field and its implementation in practice. This article starts filling this gap.

Most migration policy today is coined as restrictive or at least selective (Shachar, 2016) and not often as welcoming. In the battle for talent, a ‘welcoming’ policy can be an important asset. Referring to the fact that migrants founded 52% of start-ups created in Silicon Valley between 1995 and 2005 the European Commission’s EU Entrepreneurship 2020 Action Plan (European Commission, 2012) called for the EU member states to remove legal obstacles to the establishment of businesses by legally resident migrants and to facilitate access to information and networking prospective immigrant entrepreneurs. A not so ambitious call if you consider that migration policy is an important barrier for entrepreneurs who want to expand (Startup Genome, 2017). There is a lively battle for multinational headquarters and foreign investment going on in most western EU countries. In 2017 France ‘attracted a total of 1,298 new job-creating foreign investments […] that created or maintained 33,489 jobs.’ (Business France, 2017).^1^ The Netherlands attracted 357 foreign businesses such as Netflix, which created 12,686 jobs (NFIA, 2018). Less advertised are the numbers and the results of smaller businesses, run by individual TCNs, self-employed or small enterprises. They may not be welcomed by a foreign investment recruitment team, rolling out an ‘orange carpet’ as the Netherlands Foreign Investment Agency does.^2^ They are welcomed by immigration policies.

In this article, these immigration policies for TCN entrepreneurs are scrutinized on their level of welcoming. By presenting a first-time model for defining the level of ‘welcoming’, this study adds an instrument to the toolbox of both scholars and policy makers for evaluating migration policies. The model will be tested on the immigration policies of three Western- European countries ranked in the top-20 of the Global Innovation Index, The Netherlands, Germany and France.^3^ Three elements have been selected to define the ‘welcoming’ nature of immigrant policy: a material element, a procedural element and an institutional element. Materially, welcoming means that migration policy gives some leeway, first at the stage of admission. Welcoming means the host state is not focusing at the risks the proposed business might run, but is willing to give the applicant a chance, possibly even lend a hand. Defining the material test of the national interest in the admission of TCN entrepreneurs is central to this analysis. A second stage arrives when the immigrant applies for an extension of a residence permit or for long term residence. Welcoming policies may include accelerated options into permanent residence, possibly setting less evidentiary requirements. Procedurally, welcoming is defined as the time it takes to decide an application, relevant because an entrepreneur cannot wait too long to have the business up and running. Welcoming also includes formal criteria such as fees to be paid, transparency of the procedure and available legal protection. The third element of welcoming is related to the institutional or private actors deciding on an application. Selecting foreign investors, entrepreneurs (experienced, scale-ups or start-ups) and solo self-employed migrants is often interlinked with economic action plans. Regional strategies are important when it comes to retaining highly skilled migrants such as (former) students (David & Coenen, 2014), and may just as well be important in selecting entrepreneurs and designing entrepreneurship policy. Private actors, such as chambers of commerce or professional organizations may also play their part in defining economic needs. Thus, the question which institutional or private actors decide, or are involved in the decision-making process on admitting immigrant entrepreneurs, is the third element of the ‘welcoming talent’ model. Following from the above the research question addressed is: How welcoming are French, German and Dutch immigration policies and practices for TCN immigrant entrepreneurs?

The contribution starts with a section on methods (Methods section), followed by Migration policies selecting TCN entrepreneurs section presenting the policies on selecting TCN entrepreneurs in France, Germany and the Netherlands. The article concludes with a discussion of these policies in comparison and suggestions for the developed of a common EU entry policy for immigrant entrepreneurs.

## Methods

The analysis below draws on primary and secondary sources. An analysis of the French, German and Dutch legal documents (i.e. law, regulations and case law) on the entry of immigrant entrepreneurs is applied. Personal correspondence with legal experts on the French system added some details. Online search of the websites of German, French and Dutch Courts, allowed me to retrieve relevant case law, when available. Statistical data on the number of permits was available online for Germany and through personal correspondence with Dutch authorities. French statistics were not readily available. The Dutch case builds on an extensive case-file study commissioned by the city of Amsterdam (De Lange, 2016). The cooperation with the city lead to me being invited to workshops designing welcoming ‘customer journeys’ for immigrant entrepreneurs. These workshops led to the City Deal Warm Welcome Talent (2016), a program for improving Dutch entry policy and practice for TCN entrepreneurs. The city of Amsterdam as well as the other Dutch government agencies involved shared their ambitions with me. I participated not just as a researcher, but as a legal expert as well. These encounters can be qualified as a research-policy dialogue (Penninx, 2017) and this contribution is in a way part of that dialogue.

Germany, France and the Netherlands, (near) neighboring western European countries, have different labor markets, entrepreneurial climate and socio-economic characteristics that may well explain current diverging policies. The selected countries do share a common EU migration policy for TCN such as highly skilled employees, students and researchers, but not (yet) for immigrant entrepreneurs. This could have been different. The admission of self-employed TCN was part of a proposed Directive in 2001, but it was withdrawn as member states wanted to retain their autonomy.^4^ Member States have full discretion over immigration policies for TCN entrepreneurs.^5^ The admission of self-employed TCN is however on the EU migration agenda.^6^ Before the EU can harmonize, common denominators need to be mapped. Comparison can contribute (Boughey, 2013) to achieving the aim of convergence on the EU level. But it can also stimulate ‘legal transplants’ (Bussani & Mattei, 2012) and improve welcoming strategies in the so called ‘battle for talent’ (Shachar, 2016). “Many of the basic problems with which different countries and different legal systems have to deal are the same or similar, and often more similar than the relevant legal doctrines.” (Wilson, 2007). The challenge of being an innovative economy and welcoming TCN entrepreneurs is shared by all three countries.

## Migration policies selecting TCN entrepreneurs

The next sections investigate the welcoming nature of the immigration policies of France, Germany and the Netherlands according to the criteria listed in the ‘Welcoming Talent Model’ (Table 1). Obviously, the criteria are not exhaustive and can be expanded on in future studies.

**Table 1:** Key criteria of the welcoming talent model

Welcoming talent: material criteria	
Entry conditions (national economic interest test)	
Business plan	
Minimum investment	
Minimum job creation	
Minimum skill level	
Specific sector	
Innovative	
Relevant network	
Extension conditions	
Less evidentiary requirements	
accelerated access permanent residence	
Welcoming talent: formal criteria	
Application fees	
Length of procedure	
Transparency	
legal protection	
Welcoming talent: actors	
Public	
Regional/local	
Public-private	
Private	

### France

In 2016 France changed its migration policy for talented TCN “Enhancing the attractiveness of France by facilitating the mobility of international talent.”^7^ With this change France has introduced three different policy categories for different types of entrepreneurs. The changes to the Skills and Talents scheme are expected to be welcoming for both investors and entrepreneurs.

Prior to developing this new scheme, TCN entrepreneurs often entered under the category ‘senior executives’. This permit had to be applied for from abroad and only allowed experienced entrepreneurs to come and work and live in France. France granted 549 such senior executives permits in 2013 and 230 in 2015 (EMN, 2015). The old ‘Skills and Talent’ permit was not so welcoming for TCN entrepreneurs: they had to demonstrate that they would make a significant or lasting contribution to the French economy. The financial investment had to be at least € 300.000. The applicant had to have a portfolio that shows their entrepreneurial background, skills and education, lower than a BA was not eligible, in the specific field as well as sufficient income to support themselves while in France (EMN, 2015, p. 19). The permit did not require knowledge of the French language or set age limits (EMN, 2015, p. 19). Possibly to circumvent these requirements, applicants came as senior executives.

The new law introduces a ‘talent pass’ (*passeport talent*) which includes 10 categories of employed and self-employed TCN, including separate subcategory for investors (*l’étranger qui procède à un investissement économique direct*) and innovative entrepreneurs (*l’étranger qui justifie d’un projet économique innovant, reconnu par un organisme public*).^8^ For the investor it is not relevant what type of investment will be made or whether it’s an ‘innovative’ company that will be invested in; previous ties with France are not required either. The requirement investment is lowered from 50 to 10 jobs and from € 10 million to 500 K.^9^ Under the old scheme a central role in the admission procedure is granted to the prefect of the region where the investment is made. The prefect may lower the threshold if economic circumstances in the said department so justify. This makes sense because it is the prefect’s region where the entrepreneur will set up shop, will save or create jobs. If the entrepreneur comes along with a plan for 40 jobs the department is in dire need of, it would be unfortunate if the entrepreneur is turned down. The investor did not need an entry clearance visa for this status meaning the application can be initiated in France through the prefecture of the district where the investment is to be made.

TCN students and researchers can remain after they finish their studies in case of a business creation project in a field corresponding to their training or research.^10^ Other TCNs with a master’s degree or professional experience of at least five years of a comparable level who create a company in France based on a viable business plan and who invest at least € 30.000 are also eligible for the permit.^11^ Here it is not relevant what type of investment is made or whether it’s an ‘innovative’ company that will be started; previous ties with France are not required either it seems, but details still need to be worked out in subordinate legislation.

A new entry category for entrepreneurs is the innovative entrepreneur backed by a public organization. Startups may fall under this category. TCN applying for a startup permit must have at least a MA degree or at least five years of professional experience at a comparable level.^12^ The intended business must be realistic and serieus (*réel et sérieux*). Another option for early stage entrepreneurs is the French Tech Ticket program for international startups.^13^ Tech Ticket is a competition set up by, amongst others, the French government, that matches French incubators with top scoring foreign entrepreneurs. In 2017, 70 startups were chosen to join one of 41 incubators across several sectors. These 70 startups involve entrepreneurs from EU countries as well as from 29 different non-EU countries, with India and Brazil at the top, but also including entrepreneurs from Nigeria, Morocco, Indonesia and Canada. The startups mainly develop apps, communication and payment platforms. The startup program is unique in that it comes with a cash award (to be split between the startup and the incubator) along with the more standard benefits of incubation (work space, access to business services, expert mentoring, etc.). The startup is incubated for one-year but the duration of the residence permit is three years and at the time of renewal the entrepreneur does not need to prove that the initial company is still in existence, it is possible to achieve extension of the residence permit with a different startup company. As we will see, the program is unique compared to the programs available in the other countries in that the government actively joins in recruiting startups and does not just leave the recruitment in the hands of private players.

All Talent Passes are granted for four years, allowing for quite some time to get a business started and possible even scale up. The startup permit can be extended even if the migrant has a different startup. This shows the program accepts that failure is part of the lifecycle of a startup and it allows the migrant time to retry, instead of withdrawing the residence permit for no longer complying with the original reasons for admission.

The formal criteria include administrative fees, which are relatively low in France: for the entry visa applied for at the Consulate, the fee is € 99. The legal processing time at the Consulate for the “Passeport talent - Visa” is 90 days although in practice the visa will be available in about 1 to 3 weeks from the filing of the complete visa application.^14^ It will allow for multiple entries. Upon the entry of the applicant in France with the 3-month visa, (s)he will need to apply for a four-year residence permit for which the fee is € 269. This residence card should be issued 2 to 3 months after the application. However, in practice this processing time depends on the Prefecture where the application is filed.

To sum up, all the changes to the Skills and Talents scheme by introducing the Talent Pass are – materially speaking – expected to be favorable for both TCN investors and entrepreneurs wanting to move to France. The Tech ticket is already put in practice and received positively as an important instrument in the future economic development of France.^15^ If the procedures applied to other entrepreneurs are just as favorable is yet to be known. Questions on the transparency of the selection process in practice and what, if any, case law says on the available legal protection offered to those Talent Pass or tech ticket applicants that are refused, require time to pass since the implementation of the new scheme.

### Germany

In 2012 Germany changed its admission policy for self-employed TCNs. This change was initiated by the Sachsen government who lobbied for a change in the required €250.000 investment and the requirement to create 5 jobs. They argued that the German economy, particularly the Sachsen region, needed small- and medium enterprises (SMEs) and that the admission policy required more regional flexibility (Strunden & Pasenow, 2011). The new legislation introduced entry policy for two types of entrepreneurs, business owners (*selbständige Tätigkeit*) or professionals (*freiberufliche Tätigkeit*), also translated as freelancers.^16^ There is no national policy targeting startups. An economic needs test related to a general economic interest or to a regional need is applied on applicants of a business permit.^17^ The activities should be expected to have a ‘positive impact on the economy’ and the activities should be financed out of equity or a loan or credit facility. The assessment of economic interest is based on the sustainability of the underlying business idea, the entrepreneurial experience of the TCN, the amount of invested capital, the impact on job creation and training opportunities and the contribution to innovation and research.^18^ If the entrepreneur is older than 45 years he or she needs to provide proof of having sufficient old-age insurance, this goes for the professionals too.^19^ Professionals are for instance dentists, midwives, massage therapists, accountants, consultants, architects, journalists, artists, writers and more. If applicable they first need to have the necessary permit to practice their profession or show they have applied for it (Vollmer, 2014, p. 27).

The professionals are in the majority with 3.337 compared to the business owners, 1.630 businesses in 2016 (BAMF, 2016).^20^ In the first half of 2017 a total of 969 residence permits were granted for professionals (42 more than in the corresponding period of the previous year) and 1840 residence permits for professionals (+ 84 persons). Germany does not attract many newly arriving entrepreneurs. Of the total of 2809 people, 80.8% already held a residence permit in Germany before 2017 (BAMF, 2017).^21^ These can be TCN students who have acquired a Master Degree in Germany or TCN who work as a scientist in Germany. They can apply if the planned business activity is related to their educational background.^22^ This is a typical example of an instrument in use in many EU countries: trying to retain the TCN students and researchers who have come to the EU for studies. In 2016 out of 5.151 foreign students who switched into a permit to remain in Germany for economic reasons, only a small number obtained a permit for conducting a business (48) or as a professional (148).^23^

The Immigration Authority (*Ausländerbehörde*) has discretionary power on two levels: they *may* grant a residence permit if the conditions are met. The Immigration Authority must consult with regional Chambers of Commerce and regional and sector representatives on each application. This was to lower the standard and make it easier for immigrant entrepreneurs to qualify: the requirement of an investment of 250.000 and 5 jobs was replaced by these consultations. The amount of € 250.00 or 5 jobs is still indicative for an economic interest, but should in practice no longer be decisive.^24^ The competent bodies for the planned business location, the competent trade and industry authorities, the representative bodies for public-sector professional groups and the competent authorities regulating admission to the profession concerned shall be involved in examining the application and they consider whether the specific region has a need for the offered services or business. So, there might be regional differences as regions have different economic needs. The discretionary power of the Immigration Authorities means that they do not have to follow the regional committees’ advice, it is as the word says, an advice. The advice given by the local Chamber of Commerce is the result of an internal procedure following the application. If the applicant would want to know in advance what his chance for success would be this is not possible, says the regional government of Stuttgart.^25^ The advice is not communicated to the applicant. This means the reasoning might lack transparency and may put the applicant at a disadvantage if he were to appeal a rejection.

The regional administration *Landesamt für Bürger- und Ordnungsangelegenheiten* of Berlin provides further information and forms in English explaining in more detail the requirements.^26^ According to the forms, the following information needs to be provided by those applying for a permit to run a business: company profile, including annual turnover, main seat, number of employees. The business plan must present a ‘well-elaborated and coherent’ business concept. It should also indicate how the business will relate towards its competitors, the client target group, marketing strategy. Besides forecasts and risk prognosis the applicant must, ‘in case of innovative or totally unknown business concepts’ give additional background information (i.e. press reports, studies etc.). Capital requirements are listed, including detailed information such as license or franchise fees. This form ends with the remark ‘Many start-ups fail due to insufficient capital resources.’ So, the budget available and a revenue forecast must be presented in detail as well. Finally, the business plan must include franchise contracts, rental contracts, the purchase agreement of a business and such. This means investments must be done prior to receiving certainty on the right to a residence permit. Possibly businesses partners or banks want some certainty on this matter too before doing business with or providing financing for the migrant, creating a Catch 22 situation. Professionals, or freelancers, do not need to provide as much detailed information. Often, they must have a university degree, they need to provide their CV, financial prognosis and the license from the professional organization (Vollmer, 2014, pp. 50–52). The (private) professional organizations can thus define the welcoming nature of the entry conditions.

Case law gives us some further insights into how the economic interest test is applied. To stand a chance, knowledge of the German language is an important requirement. If the applicant does not speak German (or does not prove he does) or speaks very little English, the Chamber of Commerce is right to advise negatively.^27^ How else is the entrepreneur going to talk to his customers, business partners and the authorities? Having an interpreter is not enough. There must also be a need for the entrepreneur to be in Germany to conduct the business. A Russian entrepreneur whose GmbH was established in order to perform a wide range of activities could just as well conduct his business with temporary business visa. He planned to participate in other companies, advising on business and migration matters, public relations, export, import and services, act as real estate broker and to run a copy shop. He did not serve a national or economic interest, for his activities were investments (participation and brokering), aimed at the Russian market (advise) or provided too little jobs and investments (copy shop in Berlin). This entrepreneur had another GmbH from which he wanted to run a sushi restaurant in Berlin. The judge dryly noted that the Immigration Authorities were right to reject the application because there were already plenty of such restaurants in Berlin.^28^ Another Russian entrepreneur failed to obtain a residence permit after becoming a partner in his brothers GmbH running a hostel. He had not shown any additional activities such as further investments or expanding the business by acquiring other hostels. He had only made an investment of € 25.000, the hostels annual turnover was € 135.000 and profits were going down, lastly € 25.000 annually, making the business rather insignificant (*unbedeutend)*, according to the judge. Their town already had plenty of hotels so a regional or national economic interest was absent. The business *concept* of a Libyan transport (import and export) business specializing in transporting (unspecified) products from Libyan oil companies to Germany was not coined as insignificant as such, but the applicant failed to show plans for investments or job creation.^29^ When the appeal is lost the immigrant entrepreneur has to pay legal costs, a standard fee of € 5.000. One wonders if this money could not have been invested otherwise.

Relevant to the welcoming nature of the Germany policy is that the residence permit is granted for three years and if the entrepreneur sustains him- or herself during these three years a fast track into permanent residence is provided (which is otherwise only granted after five years), which has so far only been used by a very few entrepreneurs: In the first half of 2017 a total of 132 permanent permits were granted to entrepreneurs.^30^

On the formal issues, note the fee for handling the application ranges from € 50 to € 110, based on ‘the actual technical effort when issuing the residence permit’. A permanent permit costs € 200 but only € 100 if it is rejected. The administrations’ information doesn’t stipulate the time frame of the application but delays seem common. It has been noted that ‘language barriers, lack of legal knowledge, financing difficulties…lead to significant delays.’ (Vollmer, 2014, p. 64).

To conclude Germany seems to have a welcoming policy for highly skilled and job creating businesses operating in niche sectors. However, the low number of entrepreneurs who apply and receive, after three years, a permanent residence permit, calls for questioning the overall welcoming nature of this policy.

### The Netherlands

The Netherlands has special policies for immigrant startups, entrepreneurs and investors. The latter is hardly used and will therefore not be discussed here.^31^ The startup policy launched in January 2015 grant a visa to immigrant startup only if they have the support of an officially recognized *Startup Facilitator*.^32^ The facilitators are mostly private but can be public institutions. The facilitator must be an experienced business coach. By September 2017 the Immigration Authorities approved 34 facilitators and some 107 startups.^33^ A startup action plan must provide insight into the role of the immigrant in the startup (which must be more than an investor) and the innovative aspects of the startup. The product or service is innovative if it is new to the Netherlands, it involves new technology for production, distribution or marketing or it involves an innovative organizational set-up and working method.^34^ A specialized Economic Affairs Agency makes the assessment and advices the Immigration Authorities on this. Innovative are e.g. activities in the context of the Dutch Top Sectors which include water management, logistics and agriculture, self-developed new products or services, original approaches to sustainability problems and social innovation. The Immigration Authorities only ask Economic Affairs for an assessment if they deem the application not manifestly unfounded. The application of a Libyan entrepreneur applying for a startup visa to sell printers, import Arabic books and develop a new reading method for the blind other than braille, was deemed unfounded.^35^ According to the Court hearing his case, the fact that he teamed up with a facilitator who did not properly substantiate the application to become a *recognized* facilitator while they could have been aware of the requirements through several channels, played against him.^36^ The high evidentiary requirements are a noted obstacle (De Lange, 2016).

TCN entrepreneurs who are not startups, depend on a demonstration of their *essential economic interest* for the Netherlands through a points-based system (PBS). The PBS was introduced in 2008 to serve two purposes: “*to become more transparent and objective […] and to the governments’ policy to increase the innovative power of the Dutch economy”.*^37^ At first, very few applicants were successful. Up until 2014, only 218 entrepreneurs applied and 32 were admitted.^38^ Some failed applicants would have been successful startups. Most were ‘necessity entrepreneurs’, lower skilled migrants with no other options open (De Lange, 2016; Vance et al., 2017). The ‘City Deal Warm Welcome Talent’ (2016) hardly changed the PBS but improved the application procedures.^39^ This resulted in 134 TCN entrepreneurs admitted from 2015 till September 2017.^40^ Still, not a shocking number.

The material criteria listed in the PBS require the applicant to submit a business plan, personal experience, and – if these do not add up to enough credits – evidence of the added value for the Dutch economy. The *business plan* is used to test for the market potential of the activity. The business plan must describe ‘unique selling points’, must include a market analyses and a calculation of the cost price of the product or service to be sold. The applicant must present a financial prognosis for three years. If the immigrant entrepreneur receives a bank loan from a Dutch bank the business plan immediately gets full credits. Thus, banks can have an important say in the admission of immigrant entrepreneurs, but in practice hardly ever do (De Lange, 2016). *Personal experience* is measured by the level of education (maximum PhD) or extensive (ten years minimum) practical experience as an entrepreneur. Work experience, prior level of income and whether the applicant has a (Dutch) network also matter. This latter criterion is meant to benefit already present TCN students and researchers although half of the applicants with a migration history in the Netherlands were still rejected (De Lange, 2016). This is surprising given the fact that it’s a policy aim to keep talented migrant students, researchers and highly skilled migrant workers in the Netherlands. Those that were able to remain operated as e.g. financial advisor, chiropractor, creative designer, advisor in city ecology, intercultural publishing and webhosting. Others were transnational entrepreneurs assisting Dutch businesses in their country of origin. The importance of business experience is illustrated by the case of an Iranian trader in medical appliances. She obtained a PhD in the Netherlands and developed medical instruments, a typical university spinoff that would fit well under the startup scheme, which was not in force when she applied. Her lack of experience as an entrepreneur was held against her until one and a half years had passed and her client base and turnover had increased. The PBS also weights the *added value* to the Dutch economy: the innovative character of the activities (e.g. new on the Dutch market, a new technology, patents), the number of jobs created (a minimum of 2 fulltime jobs; high income jobs are given a higher score). Jobs created elsewhere in the EU are not considered, which could be something for EU policy makers to pick up on. The level of the investments made is relevant as well, with a minimum of € 50.000. But a high score on added value is no guarantee for a residence permit. An entrepreneur with such a high score didn’t get a residence permit. The authorities didn’t see the need for his product, a continuous on demand movie channel (a Netflix *avant la lettre)*. Likely he lost his investment of € 80.000 when he had to leave the country. This example also explains why so many other applicants do not make their intended investments while awaiting the outcome of their application. But without making the investments they do not get any points in this category. A catch-22 situation that sits bad with legal certainty and is not very welcoming.

The residence permit for a startup is granted for one year and can be extended easily if the facilitator still vouches for the startup. The residence permit for an entrepreneur is valid for two years and can be extended if the business is doing well enough. Unlike Germany, the Netherlands does not offer talented migrants accelerated access into permanent residence meaning they have to wait five years before being eligible for permanent residence. Interestingly, Dutch immigration policy does allow for hybridization: highly skilled migrant employees such as Blue Card holders may develop business activities on the side, for instance in preparation of a business spin-off.^41^

On the formal criteria it is relevant to note that the application for a startup visa does not require an entry clearance visa if the conditions for entry are met. Applicants for a residence permit as entrepreneur do require an entry clearance visa before coming to the Netherlands unless this requirement is waived due the applicants nationality or previous legal residence. Those who need the entry clearance visa are at a disadvantage. They must await the outcome of their application abroad, although short visits on the basis of a business visa might be permitted. Those who are in the Netherlands awaiting the outcome may begin doing business which will increase their chances of a successful application as for them it is easier to participate in local networks and fulfil the evidentiary requirements. It can be rather lengthy procedures, easily running up to six months. The application fee for facilitators is € 2.672 and for each individual startup residence permit € 401. The fee for entrepreneurs is € 1.336, which is considered steep.^42^ The 2016 report concluded, amongst others, that transparency on the material criteria and on the procedures was lacking. A trader in Dutch scrap metal wanted to export this for processing to Dubai. His business plan looked solid and the municipal Rotterdam Investment Agency assisted in his search for office space. He required an entry clearance visa but he did not apply for it as required. He kept traveling on temporary Schengen visas granted by other EU countries. In the end, the authorities, both local and national, dropped their support for him and the application was denied on procedural grounds. Another practical obstacle was that Economic Affairs didn’t have access to the Immigration Authorities files. Some of these practical issues have been dealt with since by improving the relevant websites and opening up direct communication between Economic Affairs and the applicant.^43^

Several institutional and private actors play a relevant role in the admission of immigrant entrepreneurs in the Netherlands. Cities can be recognized as facilitator of a startup or can assist in practical assistance when an entrepreneur is searching for a good location. Local or regional government are not involved in the decision-making process, nor are Chambers of Commerce or professional organizations. Economic Affairs and the Immigration authorities decide together and the better their cooperation, the lower the administrative obstacles. Private actors vouching for the immigrant entrepreneurs can be facilitators of startups and as such have an important say in the entry of new immigrant entrepreneurs. Banks can have an important say if the offer a loan to the immigrant entrepreneur. Here future EU policy could be of relevance, as currently the Netherlands only accepts a bank with its headquarters in the Netherlands. Banks are not keen on financing migrants without a secure residence permit though (De Lange, Besselsen, Rahouti, & Rijken, 2017) and therefor they hardly play a role in practice.

To conclude, over the past years the Netherlands has worked hard to make its admission policies for immigrant entrepreneurs more welcoming. The numbers of admitted entrepreneurs has increased. Yet little is known about the long-term residence, success and economic impact of these entrepreneurs.

## Discussion

So how welcoming are French, German and Dutch immigration policies and practices for TCN immigrant entrepreneurs? For answering this question, a ‘Welcoming Talent’ model was developed based on the material criteria, such as entry conditions, formal criteria, such as the applicable procedures and the actors involved in the decision making. Clearly, the admission policies for entrepreneurs target different types of entrepreneurs. We have seen solo self-employed, entrepreneurs providing jobs, startups, freelancing in all kinds of professions, including the arts, investors. Entrepreneurship literature shows us many more categorizations of entrepreneurs (Wright, 2011). Because migration policies do not equally cater to different types of entrepreneurs, entrepreneurs might change their realities to fit policy categories, an ‘intersection’ worth studying (Zahra & Wright, 2012), but outside the scope of this policy comparison. Also, outside the scope were multinationals opening new EU head offices, often after a strong ‘welcoming’ lobbying.^44^

Although a common EU admission policy for entrepreneurs is lacking, the EU has set some parameters in the Students & Researchers Directive 2016/801/EC. Member States shall, as of 23 May 2018, offer TCN who completed their studies or research projects in an EU Member State, a residence permit for at least nine months to set up a business (or seek employment).^45^ All three countries scrutinized allow for such a search period of twelve months. Member States may set a minimum level of the degree obtained, which minimum may be at a highly specialized level. Also, they may require that the business the researcher or student is setting up corresponds to the research or studies completed. France and Germany included this last requirement explicitly in their policy. In the Dutch PBS the level of educational attainment, Dutch diploma’s and a corresponding business plan deliver extra points. If the student is selected by one of the Dutch startup facilitators or enters the French Tech Ticket program, such criteria are no longer blocking their way. Given the fact that many facilitators for startups in the Netherlands are university based, the Dutch startup scheme can be expected to facilitate university spinoffs. Data on how many of the admitted entrepreneurs previously had a residence permit under another scheme is lacking: only Germany provides such data. In the first half of 2017 more than 80% of the entrepreneurs who applied for admission in Germany did have previous residence. The impression is that this is indeed a popular migration trajectory, although the absolute numbers are low. Because retaining foreign students and researchers is an EU policy goal the available policy instruments require fine tuning to further this aim. The recently opened up opportunity in the Netherlands which allows highly skilled migrant workers to do business on the side is welcoming because it might increase the number of business spinoffs, facilitating ‘expat-preneurs’ (Vance et al., 2017) to change status and remain in the host-state.

In all three countries ‘economic interest’ was or still is measured by a business plan, the investments made and the number of jobs created. The higher the required investment or number of jobs, the less welcoming the entry policy is, making the Dutch PBS more welcoming as it allows for compensation in other fields if no jobs are created at all or if the investment is rather low. Job creation elsewhere in the EU has been disregarded in a Dutch application. From an EU perspective this could be an interesting element to give some weight. Germany replaced the criteria based on jobs and investments by a consultation procedure and France shifted away from these criteria by opening up many other entry categories.

Self-employed professionals are a specific legal category in France and Germany. In the Netherlands the self-employed are not mentioned specifically, but a self-employed TCN isn’t necessarily excluded under the PBS. In general, the PBS seems to allow for more flexibility than the German or French system, but the new French scheme is expected to be more open than their previous investor scheme. The French Tech ticket is more welcoming than the Dutch startup scheme in respect of the funding provided, but because of that, the numbers are limited. Whether the proposed business is ‘innovative’ is measured differently. France defines innovative in relation to the tech sector while the Netherlands allows for a broader definition, including innovations in logistics and agriculture. The wider definition of what qualifies as innovative is obviously more welcoming.

The scrutinized policies seem to care only indirectly about the immigrants social and family networks, which are not listed as requirements or positive assets. On the contrary: the Dutch facilitator to a startup may not even be a family member. In literature it has been stipulated that the level of ‘embeddedness’ of the immigrant entrepreneur in ethnic, social and family networks can positively impact their success (Schutjens & Völker, 2010). The scrutinized policies neither explicitly acknowledged gendered life course differences, relevant when selecting female talent (Boucher, 2016), meaning these policies can be less welcoming for women. These are elements future immigration policies could consider more.

The welcoming model also mentions the conditions for extension of a residence permit. While the investor coming to France receives 10 years to make the investment and create the jobs an entrepreneur coming to the Netherlands has to deliver within a year. After five years in France and the Netherlands, the entrepreneur may apply for the long-term residence permit, while Germany may grant a permanent residence permit after three years. This means that an entrepreneur who makes it past the first three years in Germany is less likely to be forced to leave Germany, while entrepreneurs in the Netherlands and France need to keep their business running for at least five years to obtain a more secure residence status. Literature on startups generally accepts that if a business survives three years, it is a viable business. It would thus make sense to grant a more secure migration status after three years.

The formal criteria relate to national administrative law procedures. Fees differ greatly, with the Dutch fees obviously being the highest. The proportionality of the fees for the startup facilitator – a service provider of sorts – may be contested under EU Services Directive. The fees in Germany and France seem reasonable. The duration of procedures does not differ greatly although regional differences, especially in France and Germany with decentralized authorities, might occur. A lengthy procedure means loss of time and uncertainty on whether to start investing yes or know. Money invested might be lost if the residence permit is not granted in the end. The costs of an administrative appeal in Germany are significant, which could detract from the welcoming nature of the policies, which include access to legal protection. The schemes discussed seem relatively straight forward, but each seems to have many detailed evidentiary requirements. ‘Welcoming’ websites try to create a clear and welcoming structure for TCN entrepreneurs, which is good for transparency. The proportionality of the fees, duration of procedure, transparency on requirements and the available legal protection could be harmonized through EU policy by extending the scope of the Single Permit Directive 2011/98/EU (De Lange, 2015), already setting such procedural standards for employed migrant workers to include TCN entrepreneurs.

The key actors involved in welcoming entrepreneurs are public, regional/local, private or public-private institutions. In both France and Germany regional governments play a role of importance in the selection process. The French prefecture was permitted to waive the required investment or number of jobs. The role of the prefecture in the new Talent Pass legislation seems less prominent. The German laws refer explicitly to the need to consult (non)governmental bodies to advice the immigration authorities. The German regional authorities can influence the welcoming climate when asked to advice on the regional economic need for the applicants’ business. In the Netherlands however, decisions based on the PBS are taken centrally without any formal input from the regional or local level. The Dutch PBS leaves the immigration authorities some discretion to decide on whether to consult Economic Affairs, based on an assessment of the application not being manifestly unfounded. This means some possibly innovative TCN entrepreneurs, like the Arabic alternative to braille, may go unnoticed by the experts. Private actors are involved in the Dutch and French startup programs although the admission decision is not outsourced to them. The extent to which they influence the countries openness to TCN entrepreneurs in practice requires more on the ground research.

This exploratory study of three EU member states’ migration policies on the admission of non-EU entrepreneurs has presented a myriad of policy options, in part not so different from one another, but the devil is in the details. A future EU policy on welcoming TCN entrepreneurs must set standards for a large variety of entrepreneurs, allow for the economic interest to be broadly defined and have, at the least, transparent and practical procedures*.* Possibly the criteria and practices discussed in this contribution can serve as input for the developed of a common EU entry policy for immigrant entrepreneurs or stimulate some ‘legal transplants’. For certain, to know if these policies are welcoming and effective, more research on the actual experience of immigrant entrepreneurs and their contribution to the EU economy, labor market, innovation et cetera, over a longer period of time, is called for. In addition, as Kostakopoulou (Kostakopoulou, 2017) remarked in respect to the existing legal migration routes into the EU, further scientific research on the rights, such as the right to education or family reunification of immigrant entrepreneurs could aid policy making in this field. The model presented in this contribution can well be expanded to include other policies and practices influencing the welcoming of immigrant entrepreneurs.

## Abbreviations

BA: Bachelor of Science
EU: European Union
MA: Master of Science
PBS: Points Based System
TCN: Third Country National

## Footnotes

1 These data include EU investments. US headquarters of multinationals, such as Facebook, take the lead.

2 See the NFIA, Retrieved from https://investinholland.com

3 Retrieved from https://www.globalinnovationindex.org/, last visited on 30 April 2018. The Netherlands (3), Germany (10) and France (15).

4 Proposal for a Council Directive on the Conditions of Entry and Residence of TCNs for the Purpose of Paid Employment and Self –Employed Activities of July 11, 2001, COM(2001)386.

5 Small cracks in the national discretionary powers are visible: Turkish self-employed have their own regime under the EC-Turkey Association Agreement as do Long Term Residents using their intra-EU mobility rights and so do TCN students working as a self-employed person to pay for their studies.

6 The subject was raised in the 2015 European Commission Public consultation on the EU Blue Card and the EU’s labor migration policies, Retrieved from http://ec.europa.eu/dgs/home-affairs/what-is-new/public-consultation/2015/consulting_0029_en.htm. The subject will also be an issue during the 2016 Dutch Presidency, arguing harmonization of self-employed talent migration is needed for the development of the EU economy.

7 Press communication of the Council of Ministers of 23 July 2014, Retrieved from https://www.legifrance.gouv.fr/affichLoiPreparation.do?idDocument=JORFDOLE000029287359&type=general&typeLoi=proj&legislature=14

8 A new art. L313–20 has been introduced for the ‘passeport talent’ in the migration law. Retrieve from http://www.legifrance.gouv.fr/affichLoiPreparation.do?idDocument=JORFDOLE000029287359&type=general&typeLoi=proj&legislature=14. For the debates in the Assemblee, Retrieve from http://www.assemblee-nationale.fr/14/projets/pl2183.asp

9 Retrieved from http://www.legifrance.gouv.fr/affichLoiPreparation.do?idDocument=JORFDOLE000029287359&type=general&typeLoi=proj&legislature=14. For the debates in the Assemblee, Retrieve from http://www.assemblee-nationale.fr/14/projets/pl2183.asp

10 Art. L. 313–8, amendment proposed on 21 February 2018, Retrieved from http://www.assemblee-nationale.fr/15/textes/0714.asp#D_Article_20.

11 Art. L. 313–20 sub 5°. Retrieve from https://www.welcometofrance.com/en/you-are-coming-to-run-a-company-in-france, last visited 30 April 2018.

12 Art. L. 313–20 sub 5°.

13 Retrieved from http://www.frenchtechticket.com/.

14 Personal Communication with a Fragomen Lawyer dd. 28 September 2017.

15 Le Monde.fr, ‘French Tech Toulouse: Le numérique, « une bonne partie de notre avenir économique’. Retrieved from http://www.lemonde.fr/la-france-connectee/article/2017/05/02/french-tech-toulouse-le-numerique-une-bonne-partie-de-notre-avenir-economique_5120796_4978494.html#M6O8kkWUBBqRP3Kd.99

16 Art 21 par. 1 and par. 5 Augenthaltsgezet. Retrieved from http://www.gesetze-im-internet.de/englisch_aufenthg/englisch_aufenthg.html

17 Art. 21 par. 1 Aufenthaltsgezets.

18 Art. 21 par. 1 Aufenthaltsgezets.

19 Art. 21 par. 3 and 5 Aufenthaltsgezets

20 The number of successful applicants is on the rise: 2012: 3.806, 2013: 4.263, 2014: 4.573, 2015: 4.661 and in 2016: 4.967, Retrieved from http://www.bamf.de/DE/Infothek/Statistiken/Wanderungsmonitor/wanderungsmonitor-node.html).

21 BAMF Wanderungsmonitor first half of 2017, Retrieved from http://www.bamf.de/SharedDocs/Anlagen/DE/Publikationen/Broschueren/wanderungsmonitoring-halbjahr-2017.html?nn=2080452.

22 Art. 21 par 2a Aufenthaltsgezets.

23 Bundesamt für Migration und Flügtlinge, *Wanderungsmonitoring: Erwerbsmigration nach Deutschland, Bericht für das Jahr 2016*

24 Verfassungsgericht Berlin 16 November 2016, no. 14 K 240.15 V par. 24. Retrieved from http://www.gerichtsentscheidungen.berlin-brandenburg.de.

25 Retrieved from https://www.stuttgart.ihk24.de/Fuer-Unternehmen/recht_und_steuern/Arbeitsrecht/Auslaenderrecht/Selbstaendige_Taetigkeit_durch_Auslaender/684940 last visited on 25 August 2017.

26 Retrieved from https://service.berlin.de/dienstleistung/305249/en/, last visited 26 August 2017.

27 Verfassungsgericht Berlin 16 November 2016, no. 14 K 240.15 V, ECLI:DE:VGBE:2016:1116; VG14K240.15 V.0A; Verfassungsgericht Berlin 28 September 2016, no. 8 K 100.16 V; Verfassungsgericht Berlin 7 March 2016, no. 22 K 93.15 V. These three cases were the only three handed down in 2016 available on Beck online database and searched for on the Courts Website discussing the economic interest test.

28 Verfassungsgericht Berlin 28 September 2016, no. 8 K 100.16 V.

29 Verfassungsgericht Berlin 16 November 2016, no. 14 K 240.15 V, ECLI:DE:VGBE:2016:1116.

30 Art. 21 par. 4 Aufenthaltsgezets. In 2012, when the new law came into force 182 TCN entrepreneurs applied for permanent residence, 2013:167, 2014: 200, 2015: 256, 2016: 271 (BAMF 2016); BAMF 2017.

31 Retrieved from https://ind.nl/en/documents/faq_startup.pdf, retrieved 7 June 2018; By October 2016 two investor residence permit were granted. It requires an investment of € 1,250,000. Retrieved from https://ind.nl/en/documents/faq_regeling_buitenlandse_investeerder_en.pdf, retrieved 7 June 2018.

32 Art. 3.30(6) Vreemdelingenbesluit [Immigration Decree], art. 3.20b Vreemdelingenvoorschrift [Ministerial immigration regulation] and par. B6/4.5 Immigration Policy Guidelines.

33 Personal communication with Economic Affairs (Netherlands Enterprise Agency) of 25 September 2017.

34 These aspects clearly answer to Schumpeter’s (1934, cited by Engelen, 2010) definition of innovation.

35 District Court The Hague 12 February 2018, ECLI:NL:RBDHA:2018, p. 1723.

36 District Court The Hague 12 February 2018, ECLI:NL:RBDHA:2018, p. 1723.

37 Parliamentary Documents 2005–2006, 29, 696, No. 3, p. 1.

38 Information provided by the INS. These statistics do not include specific categories of entrepreneurs, namely Turkish entrepreneurs applying under the EC-Turkey Association Agreement, entrepreneurs using the intra-EU mobility rights under the Long-Term residence Directive (Della Torre & De Lange, 2017) or USA nationals using the Dutch-USA Friendship Treaty. Also, the Dutch-Japanese Trade Agreement is a legal source for exempting entrepreneurs from Japan from the points-based system. Guild (2017) has discussed how EU trade agreements entered into with the Western Balkans might also provide for a less restrictive scheme.

39 Personal communication with immigration lawyers during a training (November 2017).

40 Personal communication with immigration lawyers during a training (November 2017).

41 Art. 1d Besluit Wet arbeid vreemdelingen [Decree subordinate to the Act on immigrant labour], per 1 April 2017.

42 Retrieved from https://ind.nl/Paginas/Kosten.aspx, last visited 29 April 2018.

43 A website for doing business in the Netherlands was launched early 2018: Retrieved from https://business.gov.nl/

44 The mobility of managing shareholders falls within the scope of the Intra-Corporate Transfer Directive 2014/66/EU.

45 Art. 25 Directive 2016/801/EC.
